# Causal relationship between gut microbiota with subcutaneous and visceral adipose tissue: a bidirectional two-sample Mendelian Randomization study

**DOI:** 10.3389/fmicb.2023.1285982

**Published:** 2023-10-31

**Authors:** Feng Cao, Feng Pan, Xin Gong, Wen Wang, Yanyan Xu, Pengwei Cao, Yong Wang

**Affiliations:** ^1^Department of General Surgery, University Hospital RWTH Aachen, Aachen, Germany; ^2^Department of General Surgery, The Second Affiliated Hospital of Anhui Medical University, Hefei, China; ^3^Department of General Practice, Anqing Hospital Affiliated Hospital of Anhui Medical University, Anqing, China; ^4^Department of General Surgery, The First Affiliated Hospital of Anhui Medical University, Hefei, China; ^5^Department of General Surgery, The Shenzhen Hospital of Southern Medical University, Shenzhen, China

**Keywords:** gut microbiota, subcutaneous adipose, visceral adipose, Mendelian Randomization, causal inference

## Abstract

**Background:**

Numerous studies have revealed associations between gut microbiota and adipose tissue. However, the specific functional bacterial taxa and their causal relationships with adipose tissue production in different regions of the body remain unclear.

**Methods:**

We conducted a bidirectional two-sample Mendelian Randomization (MR) study using aggregated data from genome-wide association studies (GWAS) for gut microbiota and adipose tissue. We employed methods such as inverse variance weighted (IVW), MR Egger, weighted median, simple mode, and weighted mode to assess the causal relationships between gut microbiota and subcutaneous adipose tissue (SAT) as well as visceral adipose tissue (VAT). Cochran’s Q test, MR-Egger regression intercept analysis, and MR-PRESSO were used to test for heterogeneity, pleiotropy, and outliers of the instrumental variables, respectively. Reverse MR was employed to evaluate the reverse causal relationships between SAT, VAT, and gut microbiota with significant associations.

**Results:**

IVW results demonstrated that *Betaproteobacteria* were protective factors for SAT production (OR = 0.88, 95% CI: 0.80–0.96, *p* = 0.005) and VAT production (OR = 0.91, 95% CI: 0.83–0.99, *p* = 0.030). Various bacterial taxa including *Ruminococcaceae UCG002* (OR = 0.94, 95% CI: 0.89–0.99, *p* = 0.017), *Methanobacteria class* (OR = 0.96, 95% CI: 0.92–1.00, *p* = 0.029), and *Burkholderiales* (OR = 0.90, 95% CI: 0.83–0.98, *p* = 0.012) were associated only with decreased SAT production. *Rikenellaceae RC9 gut group* (OR = 1.05, 95% CI: 1.02–1.10, *p* = 0.005), *Eubacterium hallii group* (OR = 1.08, 95% CI: 1.01–1.15, *p* = 0.028), *Peptococcaceae* (OR = 1.08, 95% CI: 1.01–1.17, *p* = 0.034), and *Peptococcus* (OR = 1.05, 95% CI: 1.00–1.10, *p* = 0.047) were risk factors for SAT production. Meanwhile, *Eubacterium fissicatena group* (OR = 0.95, 95% CI: 0.91–0.99, *p* = 0.019), *Turicibacter* (OR = 0.93, 95% CI: 0.88–0.99, *p* = 0.022), and Defluviitaleaceae UCG011 (OR = 0.94, 95% CI: 0.89–0.99, *p* = 0.024) were protective factors for VAT production. Furthermore, *Bacteroidetes* (OR = 1.09, 95% CI: 1.01–1.17, *p* = 0.018), *Eubacterium eligens group* (OR = 1.09, 95% CI: 1.01–1.19, *p* = 0.037), Alloprevotella (OR = 1.05, 95% CI: 1.00–1.10, *p* = 0.038), and *Phascolarctobacterium* (OR = 1.07, 95% CI: 1.00–1.15, *p* = 0.042) were associated with VAT accumulation. Additionally, reverse MR revealed significant associations between SAT, VAT, and *Rikenellaceae RC9 gut group* (IVW: OR = 1.57, 95% CI: 1.18–2.09, *p* = 0.002) as well as *Betaproteobacteria* (IVW: OR = 1.14, 95% CI: 1.01–1.29, *p* = 0.029), both acting as risk factors. Sensitivity analyzes during bidirectional MR did not identify heterogeneity or pleiotropy.

**Conclusion:**

This study unveils complex causal relationships between gut microbiota and SAT/VAT, providing novel insights into the diagnostic and therapeutic potential of gut microbiota in obesity and related metabolic disorders.

## Introduction

In recent years, the escalating prevalence of overweight and obesity has led to a dramatic increase in the risk of obesity-related metabolic disorders (Afshin et al., 2017; Bluher, 2019). This phenomenon is believed to be rooted in the aberrant accumulation of adipose tissue within the human body. Adipose tissue is now widely recognized as a pivotal endocrine organ, releasing free fatty acids (FFAs), inflammatory cytokines, and adipokines that are intricately linked to a range of diseases including cardiovascular ailments, diabetes, renal dysfunction, and cancer (Kumari et al., 2019). Differentiated by their anatomical distribution, adipose tissue predominantly manifests as subcutaneous adipose tissue (SAT) and visceral adipose tissue (VAT). Despite both being classified as white adipose tissue, their cellular morphology and functions exhibit significant discrepancies. Notably, VAT boasts a richer vascular and neural network and higher cellular metabolic activity in comparison to SAT. For instance, studies by Dou et al. have highlighted VAT’s heightened responsiveness to catecholamine induction and substantially elevated FFA release in contrast to SAT (Dou et al., 2020). However, VAT displays lower insulin sensitivity, rendering it a principal driver of dyslipidemia and insulin resistance in obese individuals (Vajravelu et al., 2022). Moreover, VAT exhibits increased expression of cellular factors such as IL-6, TNF-α, and adiponectin (Ohman et al., 2009). In contrast, SAT showcases greater responsiveness to adiponectin’s lipolytic effects and higher secretion of leptin (Tinggaard et al., 2017). While numerous investigations have confirmed distinct behaviors of SAT and VAT in various diseases (Sam, 2018; Brusatori et al., 2022), the precise regulatory mechanisms governing these differences remain elusive.

An expanding body of research underscores the pivotal role of gut microbiota as a regulatory hub within mammalian adipose tissue, influencing adipocyte development and function. Studies have demonstrated that conventional mice possess 42% more total body fat than germ-free (GF) mice, and upon transplanting gut microbiota from conventional mice to GF mice, the latter experienced a 60% increase in body fat content within 2 weeks (Backhed et al., 2004). Additionally, marked differences in levels of free fatty acids (FFAs), cholesterol, and leptin have been observed between conventional and GF mice (Le Roy et al., 2019; Aron-Wisnewsky et al., 2021). Clinical investigations have further illuminated the significant compositional and abundance disparities in gut microbiota between obese individuals and those of normal weight, characterized by heightened *Firmicutes* and reduced *Bacteroidetes* abundance in the microbiota of obese subjects (Gomes et al., 2018). Notably, while the scope of existing gut microbiota research has primarily centered on the overarching adipose tissue milieu, investigations into distinctions between VAT and SAT have yet to be reported. Moreover, the diverse array of gut microbial species and their precise functional roles remain shrouded in uncertainty.

Mendelian Randomization (MR) is an approach utilizing Genome-Wide Association Study (GWAS) databases to investigate causal relationships between specific genetic variations and exposure outcomes. It possesses the advantage of being less susceptible to confounding external factors and adhering to genetic causality (Burgess et al., 2013). With the augmentation of GWAS data encompassing diverse exposure factors, MR analysis has gained widespread application across various disease investigations, including autoimmune disorders (Xu et al., 2021), neuropsychiatric conditions (Vaucher et al., 2018), and neoplasms (Long et al., 2023). Consequently, within this study, we employed bidirectional MR analysis, employing gut microbiota, VAT, and SAT as exposure factors and exposure outcomes. Our endeavor aimed to unravel the causal nexus between gut microbiota and VAT/SAT generation, alongside the delineation of specific functional bacterial taxa. A nuanced comprehension of gut microbiota’s mechanistic role within distinct adipose tissue regions augments our understanding of pathogenic mechanisms and potential diagnostic and therapeutic strategies for a spectrum of ailments.

## Methods

### Data sources

The dataset for gut microbiota composition-associated single-nucleotide polymorphisms (SNPs) was obtained from a large-scale GWAS. This comprehensive investigation comprised a cohort of 18,340 individuals primarily of European and American descent. The analysis relied on 16S ribosomal RNA gene sequencing to explore the interaction between chromosomal genetic variations and gut microbiota composition (Kurilshikov et al., 2021). This dataset encompassed a total of 211 taxa, spanning 9 phyla, 16 classes, 20 orders, 35 families, and 131 genera (Supplementary Table S1). Additionally, the outcome data of abdominal fat tissue data were also from a large-scale GWAS summary cohort which from UK Biobank dataset. The study encompassed abdominal magnetic resonance imaging (MRI) scans and a genome-wide association study in 32,860 participants of European ancestry. And segmented estimated the SAT and VAT generation using neural-network based methods from the Dixon segmentation (Liu et al., 2021).

### Instrumental variable selection

To identify instrumental variables (IVs) associated with gut microbiota, we applied the following criteria. First, the threshold for SNPs associated with individual genera was set below the genome-wide significance threshold (*p* < 5 × 10^−6^). Second, we used R^2^ < 0.001 and a clumping distance of 10,000 kb as parameters to assess linkage disequilibrium (LD) between SNPs. Third, we excluded SNPs with allele inconsistencies between exposure and outcome were excluded. Fourth, we removed palindromic and ambiguous SNPs. Fifth, we utilized the Phenoscanner v2 database^1^ to identify and exclude SNPs associated with confounding factors. The main confounding factors for adipose production were obesity, body mass index (BMI), Carbohydrate diet total cholesterol and triglycerides. Furthermore, we assessed the strength of association for each SNP by speculating F statistics using the formula: *F* = (*R*^2^ / (1−*R*^2^)) * (*N*−*K*−1) / *K*. Here, R2 denotes the fraction of variability in the exposure, *N* indicates the sample size, and *K* stands for the count of IVs. A robust IV was considered to have an *F* statistics value ≥10, while F statistics <10 indicated weak IVs (Pierce et al., 2011).

### MR analysis

**Figure 1:**
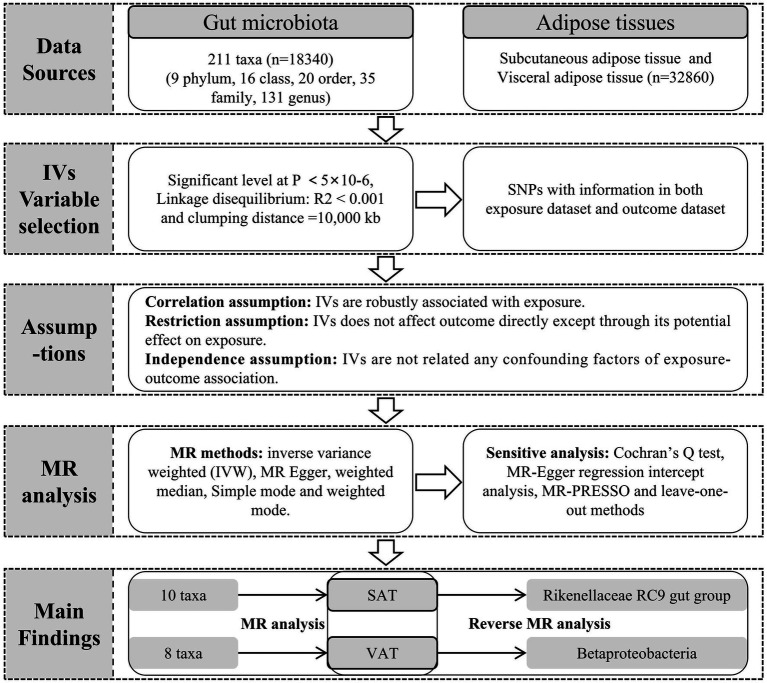
The workflow of MR analysis.

We conducted MR analysis with gut microbiota as the exposure factor and SAT and VAT production as the outcomes, satisfying the three assumptions depicted in Figure 1. During this process, a range of MR methods, including inverse variance weighted (IVW), MR Egger, weighted median, simple mode, and weighted mode, were employed to assess the causal relationships between gut microbiota and SAT/VAT production. IVW, a meta-analysis approach, combines Wald ratios from multiple SNPs. It fits a regression with the reciprocal of outcome variance as weights and disregards the intercept. In the absence of horizontal pleiotropy, IVW avoids confounding and provides unbiased estimates (Choi et al., 2019). MR Egger, on the other hand, considers the presence of an intercept term during regression and accommodates the presence of horizontal pleiotropy. When the condition of IVs strength being unrelated to the direct effect (InSIDE) is satisfied, MR-Egger regression accurately estimates causal effects (Davies et al., 2018). Weighted median estimation (WME), despite encountering less than 50% genetic variance in breach of the core MR assumption, can still accurately compute causal association effects (Kim, 2003). In cases where the InSIDE assumption is not met, the weighted mode method offers a more accurate evaluation of causal relationships compared to MR-Egger regression (Ooi et al., 2019).

### Sensitivity analysis and reverse MR analysis

To assess the potential existence of horizontal pleiotropy in IVs, we utilized both MR-Egger analysis and MR pleiotropy residual sum and outlier (MR-PRESSO) method, and *p* > 0.05 indicated no horizontal pleiotropy. In addition, MR-PRESSO was also used to test outliters. Compared with other methods, MR-PRESSO has higher accuracy and is useful in identifying horizontal pleiotropy and outliters (Verbanck et al., 2018). And if there were any outliers, we conducted a reanalysis after removing outliers. Heterogeneity was evaluated through the application of Cochran’s Q statistic, the heterogeneity being indicated when the Q value exceeds one less than the number of IVs or when *p* < 0.05 (Egger et al., 1997). Additionally, we examined the stability of causal effects through leave-one-out analysis, where individual strongly influential SNPs were excluded. Lastly, to investigate the influence of different adipose tissue regions on gut microbiota, we performed reverse MR analysis. In this analysis, SAT and VAT production were used as exposure factors, and gut microbiota was treated as the outcome, allowing us to explore the reversed causal relationships between them.

### Statistical analyzes

The study was conducted using R version 4.2.1. We utilized the software packages “TwoSampleMR (version 0.5.6)” and “MR-PRESSO (version 1.0)” for the analyzes.

## Results

### The selection of instrumental variables

We total detected 2,328 SNPs linked to 211 distinct bacterial taxa as instrumental variables (IVs) after following the criteria of *p* < 5 × 10^−6^ and *F* ≥ 10 and removing the LD effect。Among these, four microbial taxa, namely *Christensenellaceae*, *Blautia*, *Erysipelotrichaceae UCG003*, and *Lachnospira*, did not exhibit SNPs that met the specified criteria. Additionally, out of the 2,328 SNPs identified, 505 were duplicates, and 29 were associated with confounding factors. These confounding factors encompassed whole-body fat mass (e.g., rs12288512, rs182549, rs2387977), BMI (e.g., rs11109097, rs11979110, rs12894272), as well as total cholesterol (e.g., rs10108398, rs12668619, rs1530559), and triglycerides (e.g., rs11979110). The SNP count per individual bacterial taxon ranged from 5 to 22, such as, 12 SNP associated with *Betaproteobacteria* with the average F-statistic was 21.664, 11 SNP associated with *Rikenellaceae RC9 gut group* with the average F-statistic was 21.171 and 22 SNP associated with *Ruminococcaceae UCG002* with the average F-statistic was 21.413, and the details were shown in Supplementary Tables S2, S3; Table 1.

**Table 1:** Significant MR analysis results between gut microbiota and SAT generation.

GWAS ID	Bacterial taxa (exposure)	MR method	No. of SNP	F-statistic	OR	95% CI	*p*-value
GCST90016912	Betaproteobacteria	MR Egger	12	21.664	0.93	0.66–1.30	0.661
		Weighted median			0.94	0.84–1.05	0.264
		IVW			0.88	0.80–0.96	0.005
		Simple mode			0.96	0.81–1.14	0.633
		Weighted mode			0.96	0.83–1.12	0.621
GCST90017046	Rikenellaceae RC9 gut group	MR Egger	11	21.171	1.14	0.90–1.44	0.316
		Weighted median			1.05	1.00–1.11	0.051
		IVW			1.05	1.02–1.10	0.005
		Simple mode			1.07	0.98–1.17	0.139
		Weighted mode			1.07	0.99–1.16	0.122
GCST90017053	Ruminococcaceae UCG002	MR Egger	22	21.413	0.93	0.81–1.08	0.354
		Weighted median			0.93	0.86–1.01	0.869
		IVW			0.94	0.89–0.99	0.017
		Simple mode			0.91	0.77–1.06	0.224
		Weighted mode			0.90	0.78–1.05	0.192
GCST90017000	*Eubacterium hallii* group	MR Egger	16	20.975	1.14	0.99–1.31	0.088
		Weighted median			1.08	0.99–1.17	0.102
		IVW			1.08	1.01–1.15	0.028
		Simple mode			1.10	0.95–1.28	0.235
		Weighted mode			1.12	0.97–1.29	0.137
GCST90016920	Methanobacteria	MR Egger	10	22.085	0.95	0.79–1.14	0.605
		Weighted median			0.95	0.90–1.00	0.042
		IVW			0.96	0.92–1.00	0.029
		Simple mode			0.94	0.85–1.03	0.188
		Weighted mode			0.94	0.85–1.03	0.214
GCST90016942	Methanobacteriaceae	MR Egger	10	22.085	0.95	0.79–1.14	0.605
		Weighted median			0.95	0.90–1.00	0.048
		IVW			0.96	0.92–1.00	0.029
		Simple mode			0.94	0.85–1.03	0.189
		Weighted mode			0.94	0.85–1.03	0.224
GCST90016945	Peptococcaceae	MR Egger	9	22.762	1.15	0.95–1.39	0.189
		Weighted median			1.13	1.03–1.23	0.006
		IVW			1.08	1.01–1.17	0.034
		Simple mode			1.14	0.99–1.32	0.104
		Weighted mode			1.15	1.03–1.29	0.041
GCST90017042	Peptococcus	MR Egger	12	22.565	1.09	1.91–1.31	0.363
		Weighted median			1.03	0.97–1.09	0.348
		IVW			1.05	1.00–1.10	0.047
		Simple mode			1.02	0.93–1.12	0.703
		Weighted mode			1.02	0.92–1.12	0.718
GCST90017094	Burkholderiales	MR Egger	11	22.139	0.92	0.70–1.22	0.591
		Weighted median			0.92	0.82–1.03	0.130
		IVW			0.90	0.83–0.98	0.012
		Simple mode			0.94	0.78–1.12	0.482
		Weighted mode			0.95	0.79–1.14	0.581
GCST90017102	Methanobacteriales	MR Egger	10	22.085	0.95	0.79–1.14	0.605
		Weighted median			0.95	0.89–1.00	0.048
		IVW			0.96	0.92–1.00	0.029
		Simple mode			0.94	0.85–1.03	0.194
		Weighted mode			0.94	0.85–1.03	0.218

### Causal effects of gut microbiota on SAT and VAT generation

**Figure 2:**
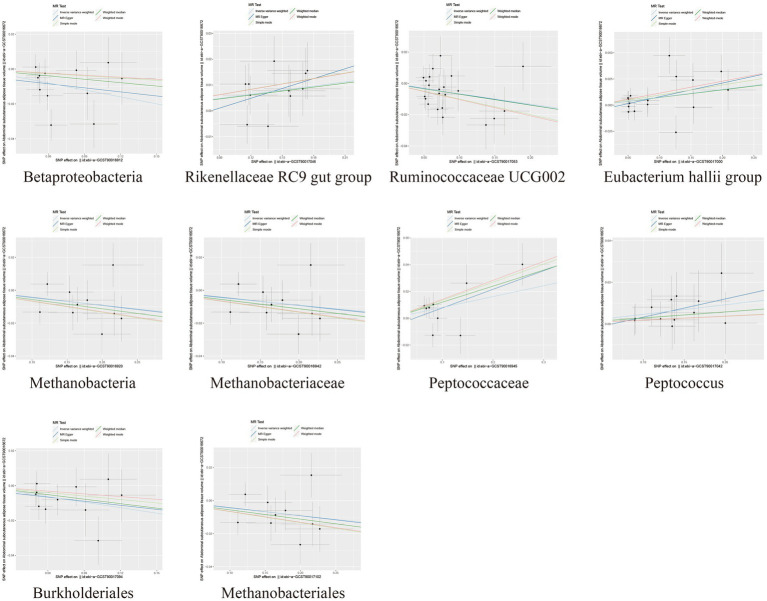
Scatter plots depicting the causal relationship between gut microbiota and SAT generation.

In the MR analysis results, 10 bacterial taxa were found to be correlated with SAT production, while 8 bacterial taxa were linked to VAT production. Interestingly, apart from *Betaproteobacteria*, the bacterial taxa associated with both SAT and VAT generation were distinct. The IVW analysis demonstrated that *Betaproteobacteria* acted as a protective factor for SAT production (OR = 0.88, 95% CI: 0.80–0.96, *p* = 0.005) and VAT production (OR = 0.91, 95% CI: 0.83–0.99, *p* = 0.030). In contrast, *Ruminococcaceae UCG002* (OR = 0.94, 95% CI: 0.89–0.99, *p* = 0.017), *Methanobacteria class* (OR = 0.96, 95% CI: 0.92–1.00, *p* = 0.029), *Methanobacteriaceae family* (OR = 0.96, 95% CI: 0.92–1.00, *p* = 0.029), *Burkholderiales* (OR = 0.90, 95% CI: 0.83–0.98, *p* = 0.012), and *Methanobacteriales order* (OR = 0.96, 95% CI: 0.92–1.00, *p* = 0.029) exhibited protective effects specifically for SAT generation. Conversely, the *Rikenellaceae RC9 gut group* (OR = 1.05, 95% CI: 1.02–1.10, *p* = 0.005), *Eubacterium hallii* group (OR = 1.08, 95% CI: 1.01–1.15, *p* = 0.028), *Peptococcaceae* (OR = 1.08, 95% CI: 1.01–1.17, *p* = 0.034), and *Peptococcus* (OR = 1.05, 95% CI: 1.00–1.10, *p* = 0.047) were identified as risk factors for SAT generation (Table 1; Figure 2; Supplementary Table S3).

**Figure 3:**
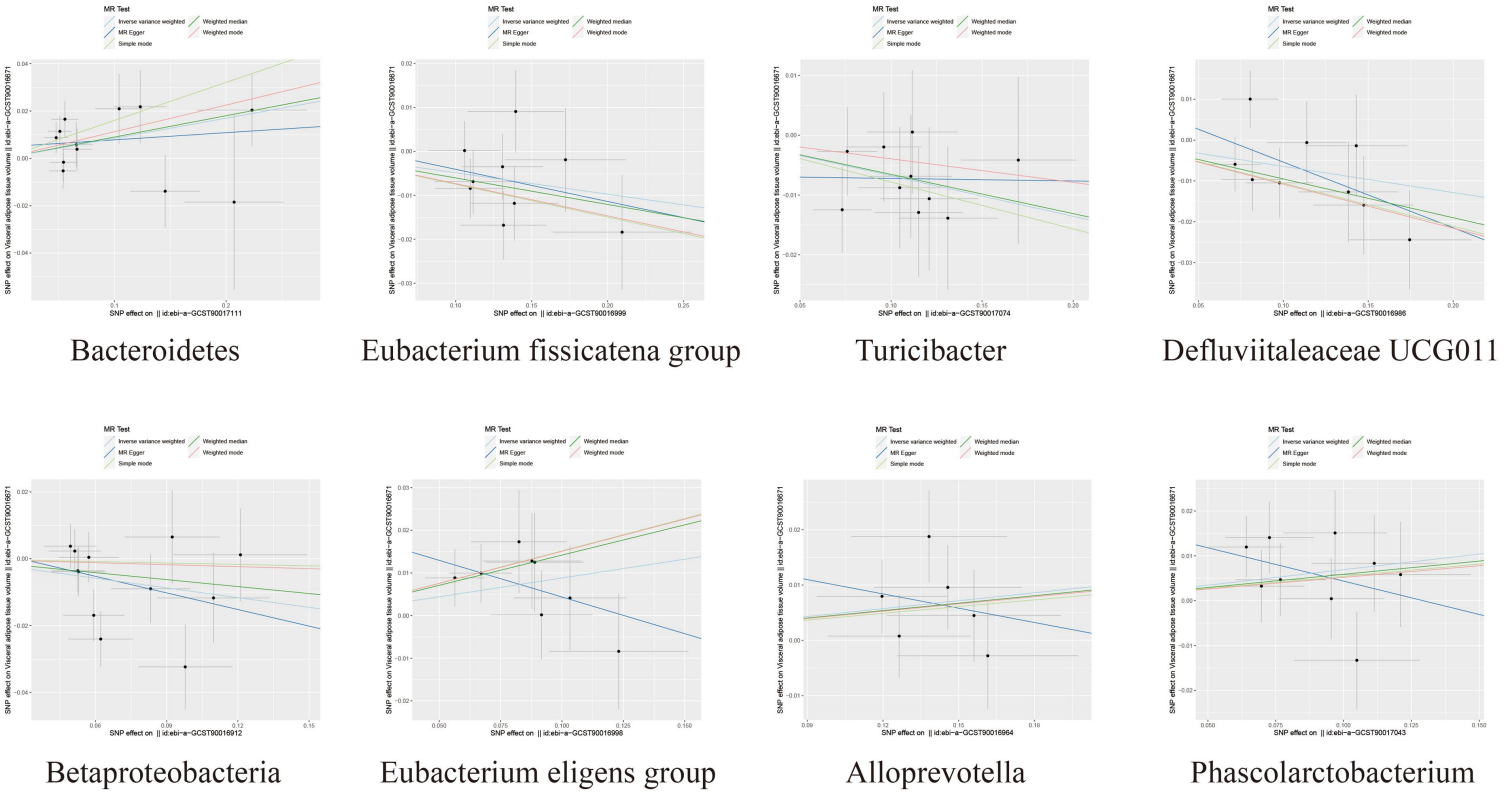
Scatter plots depicting the causal relationship between gut microbiota and VAT generation.

Among the bacterial taxa showing protective effects on VAT production, *Eubacterium fissicatena group* (OR = 0.95, 95% CI: 0.91–0.99, *p* = 0.019), *Turicibacter* (OR = 0.93, 95% CI: 0.88–0.99, *p* = 0.022), and *Defluviitaleaceae UCG011* (OR = 0.94, 95% CI: 0.89–0.99, *p* = 0.024) were notable. Conversely, bacterial taxa such as *Bacteroidetes* (OR = 1.09, 95% CI: 1.01–1.17, *p* = 0.018), *Eubacterium eligens group* (OR = 1.09, 95% CI: 1.01–1.19, *p* = 0.037), *Alloprevotella* (OR = 1.05, 95% CI: 1.00–1.10, *p* = 0.038), and *Phascolarctobacterium* (OR = 1.07, 95% CI: 1.00–1.15, *p* = 0.042) were identified as risk factors for VAT generation (Table 2; Figure 3; Supplementary Table S3). Furthermore, the results from the weighted median analysis also supported the correlations of *Methanobacteria class*, *Methanobacteriaceae family*, *Peptococcaceae*, *Methanobacteriales order*, *Eubacterium fissicatena group*, *Defluviitaleaceae UCG011*, and *Eubacterium eligens group* with both SAT and VAT.

**Table 2:** Significant MR analysis results between gut microbiota and VAT generation.

GWAS ID	Bacterial taxa (exposure)	MR method	No. of SNP	F-statistic	OR	95% CI	*p*-value
GCST90017111	Bacteroidetes	MR Egger	12	21.893	1.03	0.89–1.20	0.700
		Weighted median			1.09	0.99–1.21	0.082
		IVW			1.09	1.01–1.17	0.018
		Simple mode			1.17	0.99–1.40	0.100
		Weighted mode			1.12	0.98–1.28	0.134
GCST90016999	*Eubacterium fissicatena* group	MR Egger	9	21.157	0.93	0.75–1.16	0.533
		Weighted median			0.94	0.89–1.00	0.037
		IVW			0.95	0.91–0.99	0.019
		Simple mode			0.93	0.85–1.01	0.124
		Weighted mode			0.93	0.85–1.02	0.148
GCST90017074	Turicibacter	MR Egger	10	22.415	1.00	0.78–1.27	0.973
		Weighted median			0.94	0.87–1.01	0.070
		IVW			0.93	0.88–0.99	0.022
		Simple mode			0.92	0.82–1.04	0.215
		Weighted mode			0.96	0.86–1.08	0.523
GCST90016986	Defluviitaleaceae UCG011	MR Egger	9	22.870	0.85	0.71–1.03	0.139
		Weighted median			0.91	0.84–0.98	0.014
		IVW			0.94	0.89–0.99	0.024
		Simple mode			0.90	0.80–1.01	0.114
		Weighted mode			0.90	0.80–1.01	0.101
GCST90016912	Betaproteobacteria	MR Egger	12	21.664	0.85	0.62–1.17	0.334
		Weighted median			0.93	0.84–1.03	0.190
		IVW			0.91	0.83–0.99	0.030
		Simple mode			0.99	0.83–1.17	0.872
		Weighted mode			0.98	0.83–1.16	0.820
GCST90016998	*Eubacterium eligens* group	MR Egger	8	20.687	0.84	0.59–1.19	0.370
		Weighted median			1.15	1.03–1.28	0.010
		IVW			1.09	1.01–1.19	0.037
		Simple mode			1.16	0.98–1.38	0.125
		Weighted mode			1.16	0.98–1.38	0.124
GCST90016964	Alloprevotella	MR Egger	6	21.198	0.92	0.60–1.40	0.709
		Weighted median			1.05	0.98–1.11	0.145
		IVW			1.05	1.00–1.10	0.038
		Simple mode			1.04	0.95–1.14	0.403
		Weighted mode			1.04	0.96–1.13	0.342
GCST90017043	Phascolarctobacter-ium	MR Egger	9	22.007	0.86	0.63–1.18	0.389
		Weighted median			1.06	0.97–1.16	0.214
		IVW			1.07	1.00–1.15	0.042
		Simple mode			1.06	0.91–1.22	0.488
		Weighted mode			1.05	0.91–1.21	0.490

### The results of sensitivity analysis and reverse MR analysis

**Figure 4:**
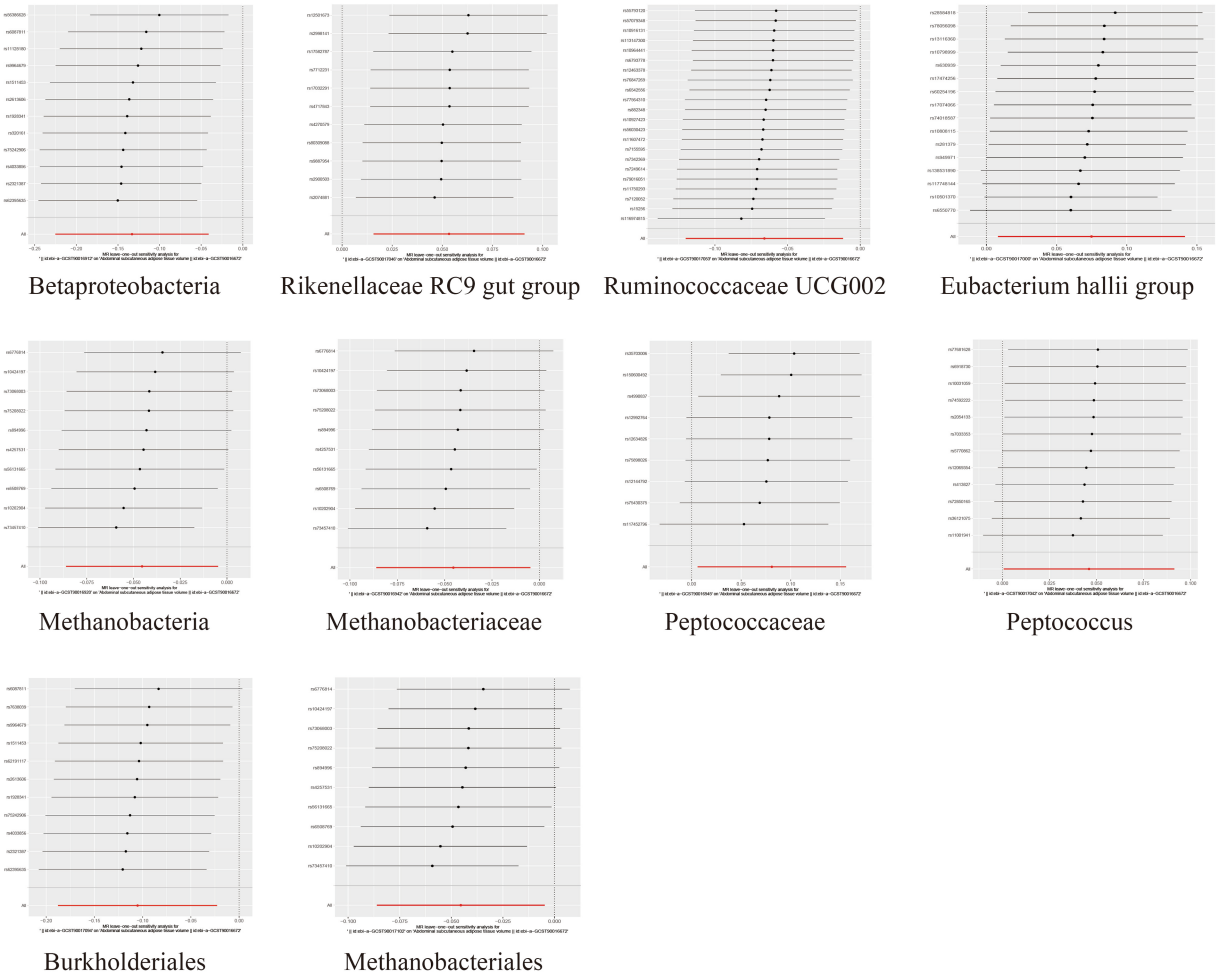
Leave-one-out diagrams illustrating the causal relationship between gut microbiota and SAT generation.

**Figure 5:**
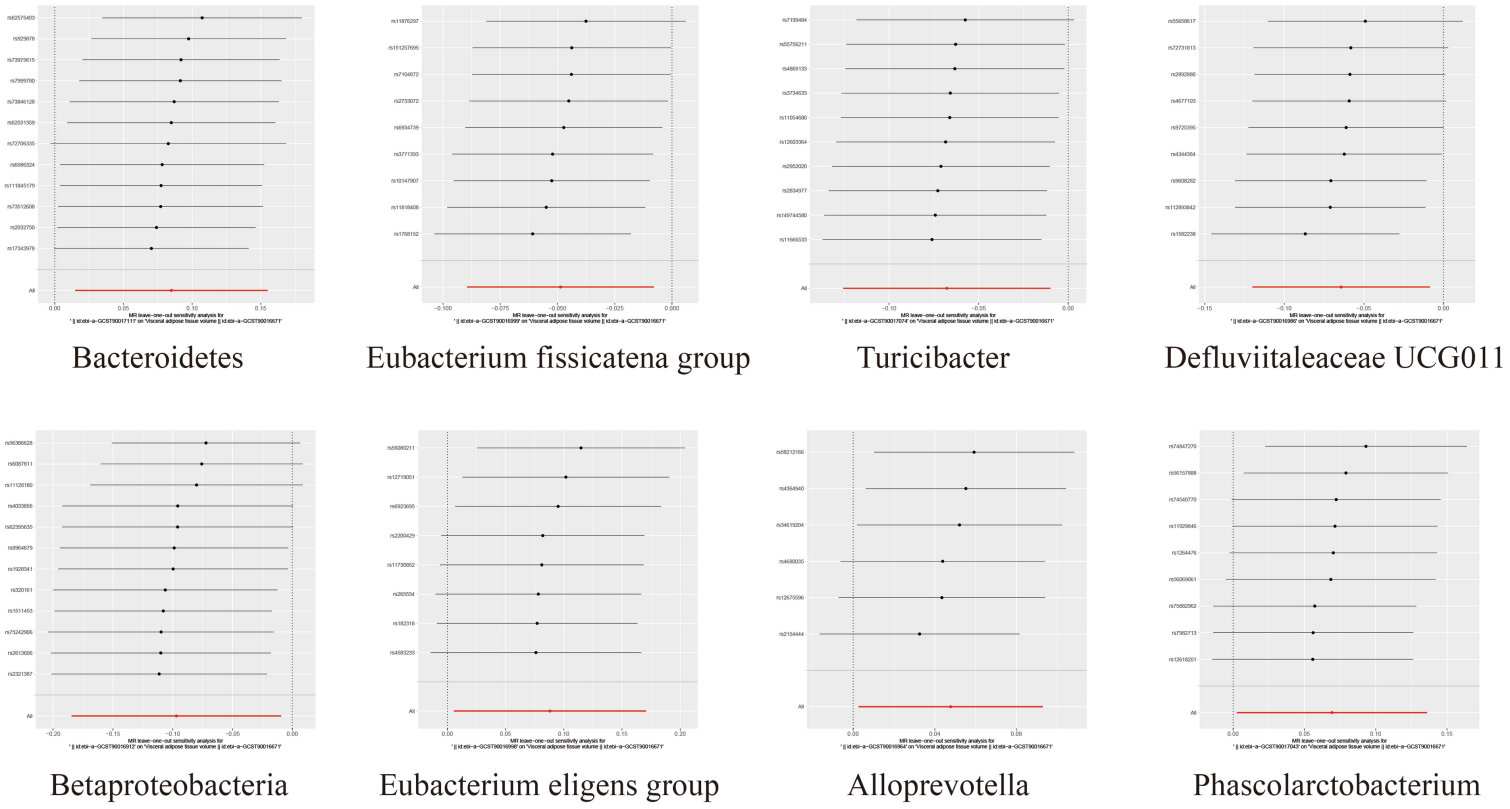
Leave-one-out diagrams illustrating the causal relationship between gut microbiota and VAT generation.

To assess the heterogeneity and pleiotropy of the MR analysis results, Cochran’s IVW Q test indicated no significant heterogeneity for the IVs related to both SAT and VAT production (*p* > 0.1) (Supplementary Tables S4, S5). The MR-Egger and MR-PRESSO analysis also did not identify the presence of horizontal pleiotropy (*p* > 0.05) (Supplementary Tables S4, S5). Moreover, leave-one-out sensitivity analysis indicated that the causal relationships between *Betaproteobacteria*, *Rikenellaceae RC9 gut group*, *Ruminococcaceae UCG002* and SAT/VAT generation were not driven by any individual SNP. Although there were potential outliers of the IVs of *Eubacterium hallii group*, *Methanobacteria*, *Methanobacteriaceae*, *Peptococcaceae*, *Peptococcus*, *Burkholderiales*, *Methanobacteriales* and the gut microbiota associated with VAT production (Figures 4, 5). But MR-PRESSO analysis did not find any significant outliers (global test *p* > 0.05) (Supplementary Tables S6, S7). Hence, this suggested that the impact of these atypical SNPs on the outcomes is relatively minor.

The reverse MR analysis further explored the influence of SAT and VAT generation on these bacterial taxa. The results indicated that, across different bacterial taxa, there were 18 to 28 SNPs strongly associated with SAT and VAT production, serving as IVs (Supplementary Tables S8, S9). Interestingly, the MR analysis revealed significant associations between SAT production and *Rikenellaceae RC9 gut group* (IVW: OR = 1.57, 95% CI: 1.18–2.09, *p* = 0.002), as well as between VAT generation and *Betaproteobacteria* (IVW: OR = 1.14, 95% CI: 1.01–1.29, *p* = 0.029), both acting as risk factors (Supplementary Tables S10, S11; Supplementary Figure S1). Cochran’s IVW Q test and MR-Egger and MR-PRESSO analysis indicated no significant heterogeneity or horizontal pleiotropy (Supplementary Table S12). MR-PRESSO analysis and leave-one-out analysis also showed no significant outliers (Supplementary Table S13; Supplementary Figure S2).

## Discussion

In this investigation, we employed a bidirectional two-sample MR analytical approach, utilizing data from the largest-scale GWAS databases covering gut microbiota and adipose tissue. Our investigation delved into the intricate interplay between these two entities, revealing noteworthy insights into the associations between gut microbiota and various adipose tissue regions. Specifically, our study highlights the protective role of *Betaproteobacteria* in both SAT and VAT production. However, the functional roles of other microbial taxa diverge across distinct adipose tissue regions. For SAT, bacterial taxa such as *Ruminococcaceae*, *Methanobacteria class*, *Methanobacteriaceae order*, *Burkholderiales*, and *Methanobacteriales family* have been found to exhibit protective effects. Conversely, taxa like *Rikenellaceae RC9 gut group*, *Eubacterium hallii group*, *Peptococcaceae*, and *Peptococcus* are deemed risk factors for the SAT phenotype. Shifting our focus to VAT, the *Eubacterium fissicatena group*, *Turicibacter*, and *Defluviitaleaceae UCG011* have shown protective roles, while *Bacteroidetes*, *Eubacterium eligens group*, *Alloprevotella*, and *Phascolarctobacterium* have been found to correlate with increased VAT. These findings underscore the intricate roles different microbial taxa play in the metabolism and regulation of SAT and VAT, potentially elucidating the biological mechanisms that underlie functional variations in distinct adipose tissue regions. Furthermore, the study also introduces a novel perspective that SAT and VAT may exert reverse effects on the abundance and functionality of specific microbial taxa. Particularly noteworthy is the potential regulation of the abundances and functions of the R*ikenellaceae RC9 gut group* and *Betaproteobacteria* by SAT and VAT, respectively.

In prior research, gut microbiota has emerged as a pivotal environmental factor influencing organism homeostasis, adipocyte accumulation, and secretion (Backhed et al., 2004), and has been associated with various diseases, including obesity (Lone et al., 2018), diabetes (Sharma and Tripathi, 2019), and Alzheimer’s disease (Chen et al., 2022). Lee et al. (2020) revealed an alteration in the diversity of gut microbiota in patients with obesity accompanied by nonalcoholic fatty liver disease (NAFLD), pinpointing *Ruminococcaceae* and *Veillonellaceae* as major bacterial taxa linked to the severity of liver fibrosis in these patients. Notably, *Ruminococcaceae* exhibited enrichment in the gut microbiota of lean mice compared to obese mice, positively correlating with high-density lipoprotein-cholesterol (HDL-C) levels and inversely correlating with serum total cholesterol (TC), and triglycerides (TGs) levels (Feng et al., 2022). Substantiating these findings, a cross-sectional study confirmed the enrichment of the *Ruminococcaceae family* in the intestines of overweight and obese individuals with weight loss (Yu et al., 2022), akin to *Bacteroidetes*. The latter is a prevalent pathogenic bacterium in the gut, and the reduced ratio of *Firmicutes* to *Bacteroidetes* constitutes a prominent feature of gut microbiota alterations in obesity (Indiani et al., 2018).

Moreover, *Betaproteobacteria*, the *Eubacterium fissicatena group*, and *Defluviitaleaceae* exhibit particularly responses to high-fat diets, exhibiting strong associations with host obesity and obesity-related metabolic disruptions (Vrieze et al., 2012; Song et al., 2021; Zhang et al., 2021). Intriguingly, some studies suggest that probiotics might attenuate the beneficial effects of *Betaproteobacteria* on body weight (Kaczmarczyk et al., 2022). Fu et al. (2021), in their investigation of banana-resistant starch (BRS) functionality, discovered that BRS improved fat accumulation by inhibiting the proliferation of *Turicibacter*. In contrast to *Ruminococcaceae*, *Turicibacter* exhibited positive correlations with TGs, TC, leptin, and insulin, possibly indicating distinct roles in regional fat regulation. Conversely, *Eubacterium hallii*, *Peptococcus*, *Eubacterium eligens group*, *Alloprevotella*, and *Phascolarctobacterium* displayed elevated abundances in the intestines of individuals with obesity or obesity-related metabolic disorders (Indiani et al., 2018; Atzeni et al., 2022; Lopez-Montoya et al., 2022; Zhao et al., 2022). Research has highlighted the similarity of *Eubacterium hallii* to *Firmicutes* species, promoting adipose tissue storage (Indiani et al., 2018). Moreover, Udayappan et al. (2016) found that treating obese diabetic mice with *Eubacterium hallii* increased fecal butyrate concentration and altered bile acid metabolism, consequently enhancing insulin sensitivity. Intriguingly, *Eubacterium eligens* and *Eubacterium hallii* have been implicated in mitigating preeclampsia and eclampsia by reducing visceral adipose tissue (VAT) accumulation, possibly linked to metabolites such as bile acids (BAs), short-chain fatty acids (SCFAs), and glutamine (Chang et al., 2020; Nie et al., 2020). Regrettably, investigations regarding *Burkholderiales*, *Methanobacteria class*, and *Peptococcaceae* in the context of fat accumulation or obesity remain unreported, warranting further exploration.

BAs, SCFAs, and glutamine, among bacterial metabolic products, are regarded as essential mediators of the bidirectional crosstalk between gut microbiota and the host (Bouter et al., 2017). Studies have indicated that a diet high in BAs can accelerate the accumulation of VAT in large animals, potentially leading to atherosclerosis and NAFLD (Yamada et al., 2017). Within the body, the BA pool comprises primary BAs synthesized in the liver and secondary BAs formed by bacterial synthesis. Nevertheless, variations in bacterial activity towards glycine or taurine conjugation of BAs contribute to disparities in secondary BA production. Notably, bile salt hydrolase activity is conserved among *Firmicutes*, *Bacteroidetes*, and *Actinobacteria* (Foley et al., 2021).

Interestingly, *Firmicutes* and *Bacteroidetes* are prominent producers of SCFAs. Comprising acetate, propionate, and butyrate, SCFAs constitute the ultimate metabolic products of the human gut microbiota. SCFAs are strongly associated with SAT. Research by Li et al. indicates that SCFAs promote SAT formation through their influence on adipocyte differentiation and metabolism (Li et al., 2014). Within this study, *Ruminococcaceae*, *Eubacterium*, *Defluviitaleacea*, and *Turicibacter* are also dominant SCFA-producing bacteria.

Furthermore, tryptophan metabolites are deemed beneficial amino acids capable of mitigating VAT accumulation. Olaniyi et al. found that tryptophan supplementation reduced VAT generation and ameliorated glucose-lipid metabolic disturbances by inhibiting adenosine deaminase/xanthine oxidase (ADA/XO) activity and enhancing glucose-6-phosphate dehydrogenase (G6PD) antioxidant capacity (Olaniyi and Olatunji, 2020). Tryptophan metabolites primarily stem from the fermentation of dietary amino acids by select bacteria, including *Bacteroides*, *Turicibacter*, and *Bifidobacterium* (Lev, 1980; Choo et al., 2017). Consequently, these pathways may underlie the functional disparities between the gut microbiome and distinct adipose tissue regions. However, the specific mechanisms governing the interplay between individual microbial taxa and SAT/VAT necessitate further exploration.

Nonetheless, this study has several limitations. Firstly, as the data are derived from aggregated sources rather than raw datasets, subgroup analyzes cannot be performed to explore causal relationships between gut microbiota and adipose tissue in different regions of the body. Secondly, the gut microbiota GWAS data primarily originates from European and American populations, with participants contributing adipose tissue data being of European descent. This potential ethnic bias could influence the generalizability of findings. Thirdly, the lowest taxonomic level for gut microbiota data is at the genus level, which constrains the granularity of specific bacterial investigations. Lastly, since comprehensive data on subcutaneous limb fat, thoracic fat, and pelvic fat are currently lacking, the SAT and VAT included in this study specifically pertain to abdominal subcutaneous fat and intra-abdominal fat, respectively. This may introduce inherent biases into the results.

## Conclusion

In summary, this study unveils intricate causal associations between the gut microbiome and distinct regional adipose tissue depots. Moreover, potential functional bacterial taxa that could influence SAT and VAT production have been identified. These findings offer novel insights for investigating the diagnostic and therapeutic potential of the gut microbiome in obesity and its related metabolic disorders. However, the mechanisms by which these microbial communities exert their effects on SAT and VAT generation remain elusive and warrant further investigation.

## Data availability statement

The datasets presented in this study can be found in online repositories. The names of the repository/repositories and accession number(s) can be found in the article/Supplementary material.

## Author contributions

FC: Conceptualization, Methodology, Writing – original draft. FP: Conceptualization, Methodology, Writing – review & editing. XG: Software, Validation, Visualization, Writing – review & editing. WW: Data curation, Formal analysis, Writing – review & editing. YX: Funding acquisition, Investigation, Supervision, Writing – review & editing. PC: Project administration, Resources, Visualization, Writing – review & editing. YW: Funding acquisition, Project administration, Supervision, Writing – review & editing.

## Footnotes

^1^ http://www.phenoscanner.medschl.cam.ac.uk/
