# Supplementary material for: Causal relationship between gut microbiota with subcutaneous and visceral adipose tissue: a bidirectional two-sample Mendelian Randomization study
Source: Front Microbiol. 2023 Oct 31;14:1285982. doi: 10.3389/fmicb.2023.1285982 (PMC10644100; doi:10.3389/fmicb.2023.1285982)
Supplement: Supplementary file 1 [file Data_Sheet_1.ZIP › Supplementary files/Figure S2.pdf]

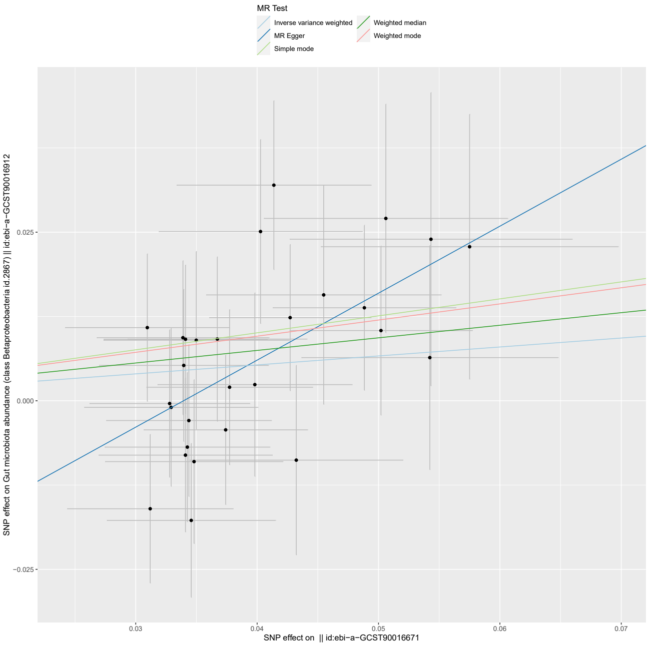
MR Test
Inverse variance weighted
MR Egger
Simple mode
Weighted median
Weighted mode
SNP effect on Gut microbiota abundance (class Betaproteobacteria id.2867) || id:ebi-a-GCST90016912
0.025
0.000
−0.025
0.03
0.04
0.05
0.06
0.07
SNP effect on || id:ebi-a-GCST90016671

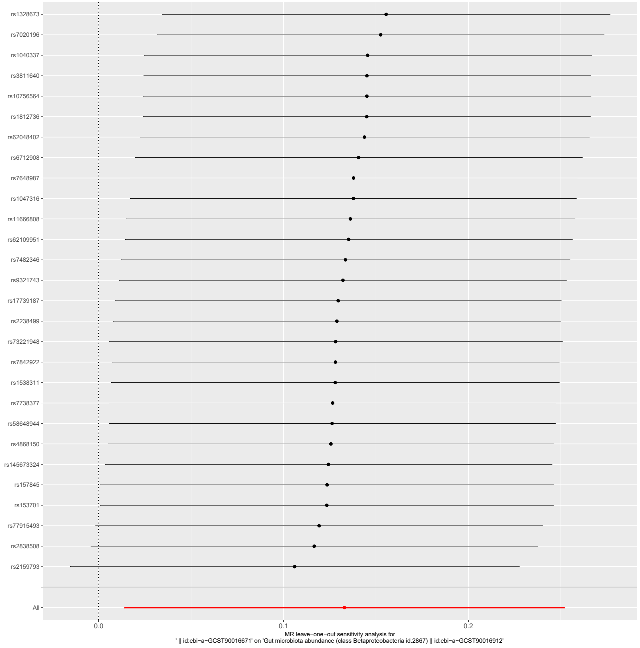
rs1328673
rs7020196
rs1040337
rs3811640
rs10756564
rs1812736
rs62048402
rs6712908
rs7648987
rs1047316
rs11666808
rs62109951
rs7482346
rs9321743
rs17739187
rs2238499
rs73221948
rs7842922
rs1538311
rs7738377
rs58648944
rs4868150
rs145673324
rs157845
rs153701
rs77915493
rs2838508
rs2159793
All
0.0
0.1
0.2
MR leave−one−out sensitivity analysis for
' || id:ebi−a−GCST90016671' on 'Gut microbiota abundance (class Betaproteobacteria id.2867) || id:ebi−a−GCST90016912'
