# Supplementary material for: Causal relationship between gut microbiota with subcutaneous and visceral adipose tissue: a bidirectional two-sample Mendelian Randomization study
Source: Front Microbiol. 2023 Oct 31;14:1285982. doi: 10.3389/fmicb.2023.1285982 (PMC10644100; doi:10.3389/fmicb.2023.1285982)
Supplement: Supplementary file 1 [file Data_Sheet_1.ZIP › Supplementary files/Table S12.docx]

**Table S11** Heterogeneity and pleiotropy results of the significant reverse MR analysis results between SAT and VAT and gut microbiota

| **Exposure** | **GWAS ID (outcome)** | **Bacterial taxa (outcome)** | **Cochran’s IVW Q test** | | | **MR-Egger intercept analysis** | | |
| --- | --- | --- | --- | --- | --- | --- | --- | --- |
|  |  |  | **Q** | **df** | ***P*-value** | **Egger intercept** | **se** | ***P*-value** |
| SAT | GCST90017046 | Rikenellaceae RC9 gut group | 15.312 | 19 | 0.703 | 0.021 | 0.031 | 0.511 |
| VAT | GCST90016912 | Betaproteobacteria | 24.815 | 27 | 0.585 | -0.004 | 0.014 | 0.234 |
