# Supplementary material for: Causal relationship between gut microbiota with subcutaneous and visceral adipose tissue: a bidirectional two-sample Mendelian Randomization study
Source: Front Microbiol. 2023 Oct 31;14:1285982. doi: 10.3389/fmicb.2023.1285982 (PMC10644100; doi:10.3389/fmicb.2023.1285982)
Supplement: Supplementary file 1 [file Data_Sheet_1.ZIP › Supplementary files/Table S13.docx]

**Table S12** MR-PRESSO analysis for significant reverse MR analysis results

| **Exposure** | **GWAS ID** | **Bacterial taxa (outcome)** | **Causal Estimate** | **SD** | **T** | ***P*-value** | **RSS_obs_** | **Global test *P*-value** |
| --- | --- | --- | --- | --- | --- | --- | --- | --- |
| SAT | GCST90017046 | Rikenellaceae RC9 gut group | 0.273 | 0.147 | 1.853 | 0.077 | 29.697 | 0.261 |
| VAT | GCST90016912 | Betaproteobacteria | 0.129 | 0.052 | 2.459 | 0.020 | 27.797 | 0.705 |
