# Supplementary material for: Causal relationship between gut microbiota with subcutaneous and visceral adipose tissue: a bidirectional two-sample Mendelian Randomization study
Source: Front Microbiol. 2023 Oct 31;14:1285982. doi: 10.3389/fmicb.2023.1285982 (PMC10644100; doi:10.3389/fmicb.2023.1285982)
Supplement: Supplementary file 1 [file Data_Sheet_1.ZIP › Supplementary files/Table S4.docx]

**Table S3** Heterogeneity and pleiotropy results of the significant MR analysis results between gut microbiota and SAT

| GWAS ID | Bacterial taxa (exposure) | Cochran’s IVW Q test | | | MR-Egger intercept analysis | | |
| --- | --- | --- | --- | --- | --- | --- | --- |
|  |  | Q | df | *P*-value | Egger intercept | se | *P*-value |
| GCST90016912 | Betaproteobacteria | 14.684 | 11 | 0.197 | -0.004 | 0.011 | 0.744 |
| GCST90017046 | Rikenellaceae RC9 gut group | 7.356 | 10 | 0.692 | -0.010 | 0.017 | 0.548 |
| GCST90017053 | Ruminococcaceae UCG002 | 20.292 | 21 | 0.503 | 0.000 | 0.006 | 0.946 |
| GCST90017000 | Eubacterium hallii group | 18.297 | 15 | 0.247 | -0.005 | 0.006 | 0.386 |
| GCST90016920 | Methanobacteria | 9.552 | 9 | 0.388 | 0.000 | 0.015 | 0.965 |
| GCST90016942 | Methanobacteriaceae | 9.552 | 9 | 0.388 | 0.000 | 0.015 | 0.965 |
| GCST90016945 | Peptococcaceae | 11.280 | 8 | 0.186 | -0.006 | 0.009 | 0.525 |
| GCST90017042 | Peptococcus | 3.117 | 11 | 0.989 | -0.005 | 0.012 | 0.652 |
| GCST90017094 | Burkholderiales | 6.487 | 10 | 0.773 | -0.001 | 0.009 | 0.843 |
| GCST90017102 | Methanobacteriales | 9.552 | 9 | 0.388 | 0.000 | 0.015 | 0.965 |
