# Supplementary material for: Causal relationship between gut microbiota with subcutaneous and visceral adipose tissue: a bidirectional two-sample Mendelian Randomization study
Source: Front Microbiol. 2023 Oct 31;14:1285982. doi: 10.3389/fmicb.2023.1285982 (PMC10644100; doi:10.3389/fmicb.2023.1285982)
Supplement: Supplementary file 1 [file Data_Sheet_1.ZIP › Supplementary files/Table S5.docx]

**Table S4** Heterogeneity and pleiotropy results of the significant MR analysis results between gut microbiota and VAT

| GWAS ID | Bacterial taxa (exposure) | Cochran’s IVW Q test | | | MR-Egger intercept analysis | | |
| --- | --- | --- | --- | --- | --- | --- | --- |
|  |  | Q | df | *P*-value | Egger intercept | se | *P*-value |
| GCST90017111 | Bacteroidetes | 11.525 | 11 | 0.400 | 0.005 | 0.006 | 0.430 |
| GCST90016999 | Eubacterium fissicatena group | 6.760 | 8 | 0.563 | 0.003 | 0.015 | 0.829 |
| GCST90017074 | Turicibacter | 2.825 | 9 | 0.971 | -0.007 | 0.013 | 0.614 |
| GCST90016986 | Defluviitaleaceae UCG011 | 7.702 | 8 | 0.463 | 0.011 | 0.010 | 0.328 |
| GCST90016912 | Betaproteobacteria | 15.729 | 11 | 0.152 | 0.005 | 0.011 | 0.670 |
| GCST90016998 | Eubacterium eligens group | 4.481 | 7 | 0.723 | 0.022 | 0.014 | 0.182 |
| GCST90016964 | Alloprevotella | 4.196 | 5 | 0.522 | 0.019 | 0.030 | 0.564 |
| GCST90017043 | Phascolarctobacterium | 7.459 | 8 | 0.488 | 0.019 | 0.014 | 0.211 |
