# Supplementary material for: Causal relationship between gut microbiota with subcutaneous and visceral adipose tissue: a bidirectional two-sample Mendelian Randomization study
Source: Front Microbiol. 2023 Oct 31;14:1285982. doi: 10.3389/fmicb.2023.1285982 (PMC10644100; doi:10.3389/fmicb.2023.1285982)
Supplement: Supplementary file 1 [file Data_Sheet_1.ZIP › Supplementary files/Table S6.docx]

**Table S5** Statistical assessment (MR-PRESSO Method) of the correlation between gut microbiota and SAT

| **GWAS ID** | **Bacterial taxa (exposure)** | **MR Analysis** | **Causal Estimate** | **SD** | **T** | ***P*-value** | **RSS_obs_** | **Global test *P*-value** |
| --- | --- | --- | --- | --- | --- | --- | --- | --- |
| GCST90016912 | Betaproteobacteria | MR-PRESSO | -0.133 | 0.047 | -2.833 | 0.016 | 17.453 | 0.270 |
| GCST90017046 | Rikenellaceae RC9 gut group | MR-PRESSO | 0.053 | 0.016 | 3.239 | 0.009 | 8.833 | 0.727 |
| GCST90017053 | Ruminococcaceae UCG002 | MR-PRESSO | -0.066 | 0.027 | -2.428 | 0.024 | 22.370 | 0.532 |
| GCST90017000 | Eubacterium hallii group | MR-PRESSO | 0.075 | 0.034 | 2.202 | 0.044 | 20.677 | 0.258 |
| GCST90016920 | Methanobacteria | MR-PRESSO | -0.045 | 0.021 | -2.188 | 0.056 | 11.926 | 0.460 |
| GCST90016942 | Methanobacteriace-ae | MR-PRESSO | 0.045 | 0.021 | -2.188 | 0.056 | 11.926 | 0.457 |
| GCST90016945 | Peptococcaceae | MR-PRESSO | 0.081 | 0.038 | 2.117 | 0.067 | 14.715 | 0.222 |
| GCST90017042 | Peptococcus | MR-PRESSO | 0.046 | 0.012 | 3.736 | 0.003 | 3.754 | 0.985 |
| GCST90017094 | Burkholderiales | MR-PRESSO | -0.105 | 0.034 | -3.104 | 0.011 | 7.886 | 0.787 |
| GCST90017102 | Methanobacteriales | MR-PRESSO | -0.045 | 0.021 | -2.188 | 0.056 | 11.926 | 0.379 |
