# Supplementary material for: Causal relationship between gut microbiota with subcutaneous and visceral adipose tissue: a bidirectional two-sample Mendelian Randomization study
Source: Front Microbiol. 2023 Oct 31;14:1285982. doi: 10.3389/fmicb.2023.1285982 (PMC10644100; doi:10.3389/fmicb.2023.1285982)
Supplement: Supplementary file 1 [file Data_Sheet_1.ZIP › Supplementary files/Table S7.docx]

**Table S6** Statistical assessment (MR-PRESSO Method) of the correlation between gut microbiota and VAT

| **GWAS ID** | **Bacterial taxa (exposure)** | **MR Analysis** | **Causal Estimate** | **SD** | **T** | ***P***  ***-*value** | **RSS_obs_** | **Global test *P*-value** |
| --- | --- | --- | --- | --- | --- | --- | --- | --- |
| GCST90017111 | Bacteroidetes | MR-PRESSO | 0.085 | 0.036 | 2.375 | 0.037 | 13.488 | 0.471 |
| GCST90016999 | Eubacterium fissicatena group | MR-PRESSO | -0.049 | 0.019 | -2.545 | 0.034 | 8.497 | 0.571 |
| GCST90017074 | Turicibacter | MR-PRESSO | 0.068 | 0.017 | -4.097 | 0.003 | 3.485 | 0.977 |
| GCST90016986 | Defluviitaleaceae UCG011 | MR-PRESSO | -0.064 | 0.028 | -2.299 | 0.051 | 9.865 | 0.485 |
| GCST90016912 | Betaproteobacteria | MR-PRESSO | -0.097 | 0.045 | -2.168 | 0.053 | 18.616 | 0.183 |
| GCST90016998 | Eubacterium eligens group | MR-PRESSO | 0.088 | 0.034 | 2.613 | 0.035 | 5.950 | 0.737 |
| GCST90016964 | Alloprevotella | MR-PRESSO | 0.048 | 0.021 | 2.261 | 0.073 | 5.835 | 0.585 |
| GCST90017043 | Phascolarctobac  -terium | MR-PRESSO | 0.069 | 0.033 | 2.108 | 0.068 | 9.385 | 0.514 |
