# Supplementary figures and images for: Causal relationship between gut microbiota with subcutaneous and visceral adipose tissue: a bidirectional two-sample Mendelian Randomization study
Source: Front Microbiol. 2023 Oct 31;14:1285982. doi: 10.3389/fmicb.2023.1285982 (PMC10644100; doi:10.3389/fmicb.2023.1285982)

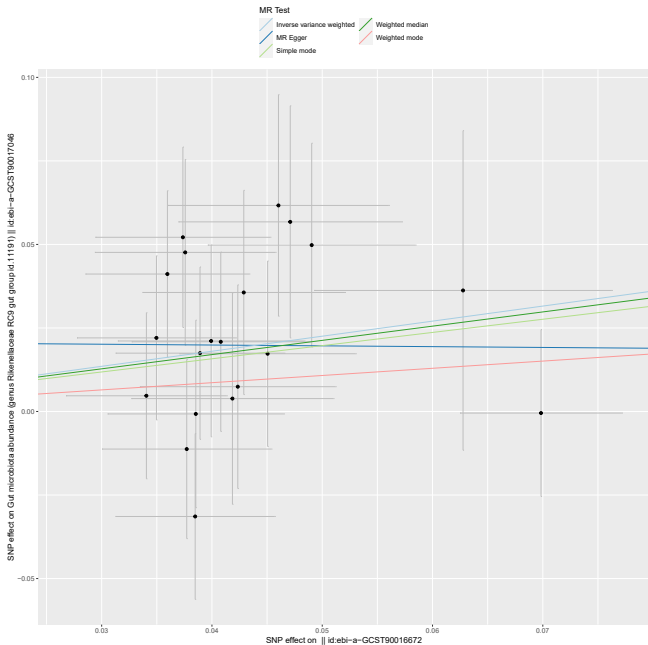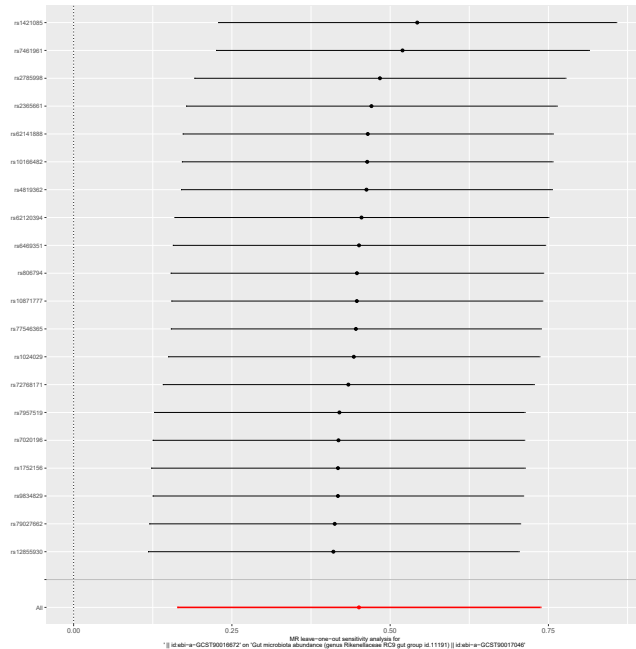

Supplement: Supplementary file 1 [file Data_Sheet_1.ZIP › Supplementary files/Figure S1.pdf]
